# Prevalence of parents' non-intention to vaccinate their children and adolescents against COVID-19: A comparative analysis in Colombia and Peru

**DOI:** 10.1016/j.jvacx.2022.100198

**Published:** 2022-07-27

**Authors:** Vicente A. Benites-Zapata, Percy Herrera-Añazco, Jerry K. Benites-Meza, Karen Bonilla-Aguilar, Diego Urrunaga-Pastor, Guido Bendezu-Quispe, Angela Uyen-Cateriano, Alfonso J. Rodriguez-Morales, Adrian V. Hernandez

**Affiliations:** a Unidad de Investigación para la Generación y Síntesis de Evidencias en Salud, Universidad San Ignacio de Loyola, Lima, Peru; b Red Internacional en Salud Colectiva y Salud Intercultural, Mexico, Mexico; c Instituto de Evaluación de Tecnologías Sanitarias en Salud e Investigación – IETSI, EsSalud, Lima, Peru; d Universidad Privada San Juan Bautista, Lima, Peru; e Sociedad Científica de Estudiantes de Medicina de la Universidad Nacional de Trujillo, Trujillo, Peru; f Grupo Peruano de Investigación Epidemiológica, Unidad de Investigación para la Generación y Síntesis de Evidencias en Salud, Universidad San Ignacio de Loyola, Lima, Peru; g Universidad Científica del Sur, Lima, Peru; h Universidad Privada Norbert Wiener, Centro de Investigación Epidemiológica en Salud Global, Lima, Peru; i Medecins Sans Frontieres, Health Politics Teams, Brussels, Belgium; j Latin American Network of COVID-19 Research (LANCOVID), Pereira, Risaralda, Colombia; k Grupo de Investigación Biomedicina, Faculty of Medicine, Fundación Universitaria Autónoma de las Americas, Pereira, Risaralda, Colombia; l Health Outcomes, Policy, and Evidence Synthesis (HOPES) Group, University of Connecticut School of Pharmacy, Storrs, CT 06269, USA; m Unidad de Revisiones Sistemáticas y Metaanálisis, Guías de Práctica Clínica y Evaluaciones Tecnológicas Sanitarias, Universidad San Ignacio de Loyola (USIL), Lima, Peru

**Keywords:** COVID-19, SARS-CoV-2, COVID-19 Vaccines, Vaccination Refusal, Vaccination, Child, Adolescent, Parents, Colombia, Peru

## Abstract

We aimed to estimate the prevalence and factors associated with parents’ non-intention to vaccinate their children and adolescents against COVID-19 in Colombia and Peru. We performed a secondary analysis using a database generated by the University of Maryland and Facebook (Facebook, Inc). We Included adult (18 and over) Facebook users residing in LAC who responded to the survey between May 20, and November 5, 2021. We Included sociodemographic characteristics, comorbidities, mental health, economic and food insecurity, compliance with mitigation strategies against COVID-19, and practices related to vaccination against this disease. We estimated crude (cPR) and adjusted (aPR) prevalence ratios with their respective 95 %CI. We analyzed a sample of 44,678 adults from Colombia and 24,302 from Peru. The prevalence of parents' non-intention to vaccinate their children and adolescents against COVID-19 was 7.41 % (n = 3,274) for Colombia and 6.64 % (n = 1,464) for Peru. In Colombia, age above 35 years old, compliance with physical distancing, use of masks, having economic insecurity, anxiety symptoms, having a chronic condition or more comorbidities, and being vaccinated were associated with a higher probability of vaccinating children and adolescents against COVID-19. In Peru, female gender, compliance with physical distancing, use of masks, having economic insecurity, anxiety symptoms, having a chronic condition or more comorbidities, having had COVID-19, and being vaccinated were associated with a higher probability of vaccinating children against COVID-19. Living in a town, a village, or a rural area was associated with a higher prevalence of non-intention to vaccinate children and adolescents against COVID-19. About 9 out of 10 parents in Colombia and Peru intend to vaccinate their children and adolescents against COVID-19. This intention is associated with some factors which are similar between the two countries, as well as other factors and variations among the different regions of each country.

## Highlights

• About 9 out of 10 parents in Colombia and Peru intend to vaccinate their children and adolescents against COVID-19.
• Colombia: being vaccinated, 35 to 54 years old, maintaining physical distance, using masks, having economic insecurity, anxiety, and comorbidities increased the intention of vaccinating children and adolescents.
• Peru: being vaccinated, female, maintaining physical distancing, using a mask, having economic insecurity, comorbidities, and have had COVID-19 increased the intention to vaccinate children and adolescents.
• Peru: living in a town, village or rural area was associated with reducing the intention to vaccinate children and adolescents.

## Introduction

The most cost-effective strategy for controlling the COVID-19 pandemic is to achieve global vaccination coverage of at least 70 to 90 % [1]. Vaccines against COVID-19 are not only safe but have also been shown to reduce hospitalizations, the use of ventilation, health care costs, mortality, as well as viral transmission [2], [3], [4], [5]. Although initially there was controversy about vaccination in the child population due to the relatively low incidence, severity, and limited spread of the disease in this age group, it is currently considered essential to achieve herd immunity [6]. Similarly, it is necessary to ensure the return to regular school learning and prevention against severe cases in children, as well as long-term consequences such as post-COVID-19 syndrome [7].

In October 2021, the Food and Drug Administration authorized the BioNTech vaccine for emergency use in children from 5 to 11 years of age [8]. Although this vaccine remains under surveillance and continuous monitoring, it has been demonstrated to be safe and effective in this population [9]. Nonetheless, investigations at a world level have suggested that some parents reject vaccination due to false information reported by the media [10], [11], leading to uncertainty regarding the safety and efficacy of the vaccines [12].

Although 9 out of 10 parents in Latin America and the Caribbean (LAC) intend to vaccinate their children, there are factors associated with the non-intention to vaccinate [13]. The proportion of parents who do intend to vaccinate their children is encouraging despite being one of the continents with the highest degree of misinformation and low adherence to the control recommendations of the pandemic [14]. However, this vaccination intention varies among the regions of each country, and each country has factors associated with its social determinants or its health systems.

There are differences among the regions of Peru concerning the intention of the adult population to be vaccinated against COVID-19 [15], being lower in older adults [16]. A study in Colombia showed lower vaccination acceptance in adults than in the Peruvian population [17], with important variations among its regions as in Peru [18]. These results suggest that the intention to vaccinate in different countries varies according to the sociodemographic context and age [19], [20], [21], [22], [23]. Likewise, fear, socioeconomic conditions, and institutional situations can also intervene in compliance with vaccination [24], [25].

Therefore, while some studies have evaluated the vaccination intention in LAC countries [13], there may be variations within these countries that warrant strategies to achieve adequate coverage in children and adolescents. Therefore, the objective of the present study was to evaluate the factors associated with the non-intention to vaccinate children and adolescents in Peru and Colombia and the variations of this intention among the different regions of these two countries.

## Methods

### Study design

We performed a secondary analysis using a database generated by the University of Maryland and Facebook (Facebook, Inc). Both institutions designed a survey to assess sociodemographic characteristics, comorbidities, mental health, economic and food insecurity, compliance with mitigation strategies against COVID-19, and practices related to vaccination against this disease. Since April 23, 2020, this survey has been carried out daily in more than 200 countries and in the primary language of the territory. The sampling frame for the random selection of participants Included daily Facebook users over 18 years of age from a particular region and country. The selection of the surveyed participants was random based on the sampling frame that was recalculated daily. If a Facebook user refused to participate, another was randomly invited within the sampling frame. Participants could only answer the survey once within an eight-week time frame. This survey has been used to develop previous studies [26], and the survey methodology has been described in greater detail elsewhere [27].

### Population and sample

**Fig. 1:**
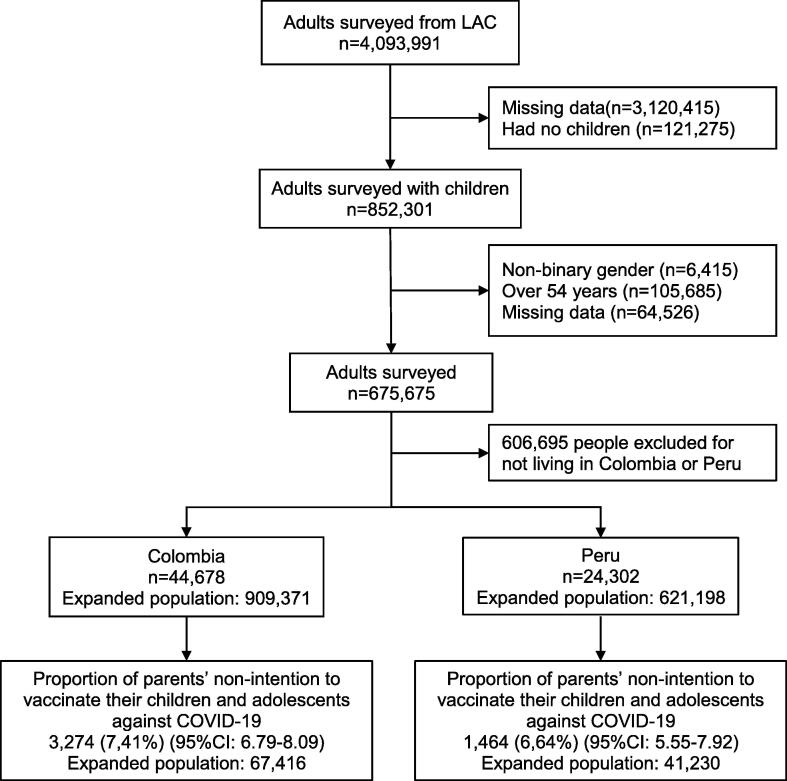
Flowchart of the study sample selection.

We included adult (18 and over) Facebook users residing in LAC who responded to the survey between May 20 and November 5, 2021. We excluded participants who did not have the variables of interest, who did not have children, those of non-binary gender, and those over 54 years of age. We excluded participants over 54 years of age to reduce the probability of not exclusively including parents of children under 18 **(**Fig. 1**)**.

### Variables and measures

**Outcome variable:** The outcome variable was parents’ non-intention to vaccinate their children and adolescents against COVID-19.

We evaluated the parents’ intention to vaccinate their children and adolescents against COVID-19 using the survey question: Will you choose to get a COVID-19 vaccine for your child or children when they are eligible? This question has four possible alternatives: yes, definitely; yes, probably; no, probably not; and no, definitely not. Subsequently, we dichotomized the variable considering the first two alternatives as to the parents’ intention to vaccinate their children and adolescents, while the last two were considered non-intention.

### Independent variables

#### Sociodemographic variables

We included the following sociodemographic variables (the survey question related to the study variables and the categories considered for these variables in the study are shown in parenthesis): gender (What is your gender?; male, female), age (What is your age?; 18 to 24 years, 25 to 34, 35 to 44, 45 to 54), educational level (What is the highest level of education that you have completed?; university post-graduate degree completed/university completed/college/pre-university, secondary school completed/high school (or equivalent) completed, primary school completed/less than primary school/no formal schooling) and area of residence (Which of the following best describes the area where you currently live?; city, town, village or rural area). Town was defined as a populated area with fixed boundaries and a local self-government; city was defined as an important or a large town; and village was defined as a group of houses and other buildings, usually in the countryside, smaller than a town.

### Comorbidities, personal and COVID-19 history

Participants self-reported the following comorbidities (survey question: Have you ever been told by a doctor, nurse, or another health professional that you have any of the following medical conditions?): asthma, chronic obstructive pulmonary disease (COPD) or chronic bronchitis or emphysema, cancer, diabetes, high blood pressure, kidney disease, compromised or weakened immune system, heart attack or another heart disease, and obesity. We generated a variable that groups the comorbidities in 0, 1, 2, or more.

We also included self-reporting of being a smoker (yes, no), having had COVID-19 (yes, no), and having been vaccinated against COVID-19 (yes, no).

### Compliance with community mitigation strategies

The community mitigation strategies included were physical distancing and mask use during the last seven days. We defined physical distancing as when a participant reported having intentionally avoided contact with other people at least at some point in the last seven days (survey question: In the past 7 days, how often did you intentionally avoid contact with other people?). In addition, the use of masks was defined if a participant reported wearing a mask in public at least at some point during the last seven days (survey question: In the past 7 days, how often did you wear a mask when in public?).

### Food and economic insecurity

We assessed food insecurity with the survey question: How worried are you about having enough to eat in the next week? This question had four possible answers: very worried, somewhat worried, not too worried, and not worried at all. We considered the first three responses as food insecurity.

We defined economic insecurity using the survey question: How worried are you about your household’s finances in the next month? There were four response alternatives: very worried, somewhat worried, not too worried, and not worried at all. We defined economic insecurity using the first three responses.

### Anxiety and depressive symptoms

We evaluated anxiety symptoms using the survey question: During the last seven days, how often did you feel so nervous that nothing could calm you down? This question is part of the Kessler Psychological Distress Scale (*K*10), and the survey has five response alternatives: all the time, most of the time, some of the time, a little of the time, and none of the time. Therefore, we dichotomized the variable considering the first four alternatives as to the presence of anxiety symptoms.

We evaluated depressive symptoms using the survey question: How often did you feel so depressed that nothing could cheer you up in the past seven days? This question is part of the *K*10 and has five response alternatives: all the time, most of the time, some of the time, a little of the time, and none of the time. Therefore, we dichotomized the variable considering the first four alternatives as to the presence of depressive symptoms.

### Statistical analysis

We downloaded the databases in Microsoft Excel 2016 ® format and imported them into the statistical package Stata/SE ® version 17.0 (StataCorp, TX, USA). Then, we performed the statistical analysis considering the complex sampling of the survey and the svy command.

We performed a descriptive analysis using absolute frequencies and weighted proportions with their respective 95 % confidence intervals (95 %CI). We used the Chi-square test with Rao-Scott correction to perform the bivariate analysis between the independent variables and the parents’ non-intention to vaccinate their children and adolescents against COVID-19. Two generalized linear models (crude and adjusted) of the Poisson family with a logarithmic link function were used to estimate the factors associated with the parents’ non-intention to vaccinate their children and adolescents against COVID-19. Crude (cPR) and adjusted (aPR) prevalence ratios with their respective 95 %CI were estimated. The adjustment for confounders was carried out according to an epidemiological approach based on other studies [13], after evaluating the collinearity of the associated factors included in the final adjusted model. We evaluated the possible collinearity of the associated factors included in the final adjusted model. A p-value<0.05 was considered statistically significant in all the analyses.

### Ethical considerations

All participants gave their informed consent before answering the survey. This study analyzed a secondary database that collected data without identifiers and did not violate the integrity of the participants. The database is not open access; the authors of this study achieved access after obtaining a signed agreement with the University of Maryland.

## Results

We analyzed a sample of 44,678 adults from Colombia and 24,302 adults from Peru **(**Fig. 1**)**.

### Colombia

#### Characteristics of the study sample in Colombia

In the Colombia study sample, 55.44 % were female, 33.19 % were between 35 and 44 years old, 53 % had completed secondary education or less, 60.88 % lived in a city, and 10.78 % reported smoking. Regarding prevention measures, 89.45 % and 92.27 % had complied with physical distancing and the use of a mask, respectively. Food insecurity was reported by 72.49 %, and 86.61 % reported having economic insecurity. In addition, 35.82 % and 42.51 % described having anxiety and depressive symptoms, respectively; 65.27 % of the participants did not report comorbidities, 47.53 % were not yet vaccinated against COVID-19, and 35.79 % had had COVID-19 at some point. In Colombia, the prevalence of parents' non-intention to vaccinate their children and adolescents against COVID-19 was 7.41 % **(**Table 1**)**.

**Table 1:** Descriptive analysis of the study samples in Colombia (n = 44,678; N = 909,371) and Peru (n = 24,302; N = 621,198).

**Characteristics**	**Colombia**			**Peru**		
	Absolute frequency of participants surveyed	Weighted proportion according to each category		Absolute frequency of participants surveyed	Weighted proportion according to each category	
	n	%	95 %CI	n	%	95 %CI
**Gender**						
Male	18,933	44.56	43.51–45.63	11,716	47.18	43.43–50.98
Female	25,745	55.43	54.37–56.49	12,586	52.81	49.02–56.57
**Age (years)**						
18–24	4,167	11.69	11.14–12.26	2,837	10.95	10.07–11.90
25–34	13,269	30.89	29.97–31.84	6,793	28.99	28.01–30.00
35–44	17,129	33.19	32.62–33.76	8,184	33.00	32.19–33.83
45–54	10,113	24.22	23.06–25.43	6,488	27.04	25.68–28.45
**Educational level**						
University post-graduate degree completed/university completed/college/pre-university	18,796	36.97	34.74–39.26	13,243	52.00	47.92–55.02
Secondary school completed/High school (or equivalent) completed	22,023	52.99	51.22–54.75	10,402	45.1	41.49–48.77
Primary school completed/Less than primary school/No formal schooling	3,859	10.03	9.23–10.90	657	3.41	2.74–4.25
**Area of residence**						
City	30,156	60.88	50.67–70.22	19,340	75.97	66.21–83.62
Town	10,578	28.68	21.63–36.95	2,793	13.24	8.67–19.73
Village or rural area	3,944	10.43	8.00–13.50	2,169	10.77	7.84–14.63
**Smoking**						
No	39,321	89.21	86.37–91.53	21,365	88.60	87.79–89.38
Yes	5,357	10.78	8.47–13.63	2,937	11.39	10.62–12.21
**Compliance with physical distancing**						
No	4,396	10.54	9.97–11.16	2,138	9.61	8.80–10.49
Yes	40,282	89.45	88.84–90.03	22,164	90.38	89.51–91.20
**Compliance with mask use**						
No	3,188	7.72	6.99–8.52	1,337	6.06	5.33–6.89
Yes	41,490	92.27	91.48–93.01	22,965	93.93	93.11–94.67
**Food insecurity**						
No	13,202	27.50	0.25–0.30	6,145	23.73	21.98–25.59
Yes	31,476	72.49	0.70–0.75	18,157	76.26	74.41–78.02
**Economic insecurity**						
No	6,558	13.38	0.12–0.15	2,603	10.04	9.43–10.70
Yes	38,120	86.61	0.85–0.87	21,699	89.95	89.30–90.57
**Anxiety symptomatology**						
No	28,196	64.17	0.63–0.65	14,040	57.68	55.88–59.47
Yes	16,482	35.82	0.35–0.36	10,262	42.31	40.53–44.12
**Depressive symptomatology**						
No	25,303	57.48	0.56–0.59	12,227	49.99	48.43–51.56
Yes	19,375	42.51	0.41–0.44	12,075	50.00	48.44–51.57
**Comorbidities**						
No	28,544	65.27	0.64–0.66	14,852	62.81	60.38–65.18
Yes	16,144	24.87	0.24–0.26	9,450	26.00	25.11–28.02
**Vaccinated**						
No	22,311	47.52	0.46–0.49	13,340	53.70	50.54–56.85
Yes	22,367	52.47	0.51–0.54	10,962	46.29	43.15–49.46
**Had COVID-19**						
No	28,949	64.20	0.62–0.66	13,438	54.08	52.14–56.02
Yes	15,729	35.79	0.34–0.38	10,864	45.91	43.98–47.86
**Parents’ intention to vaccinate their children and adolescents against COVID-19**						
Yes	41,404	92.59	91.91–93.21	22,838	93.36	92.08–94.45
No	3274	7.41	6.79–8.09	1464	6.64	5.55–7.92

95 %CI: 95 % Confidence Intervals.

#### Prevalence of parents’ non-intention to vaccinate their children and adolescents against COVID-19 according to each region of Colombia

**Fig. 2:**
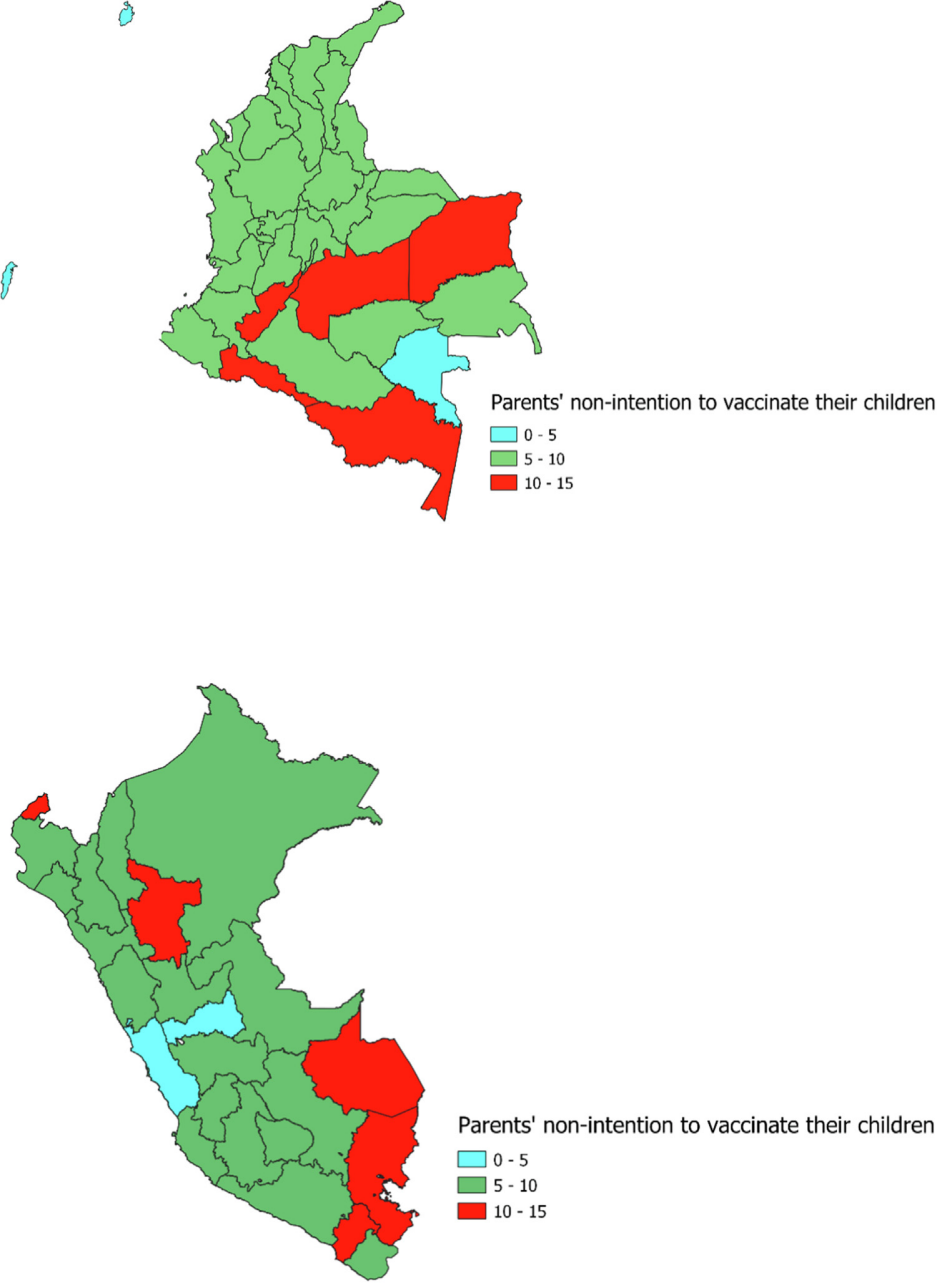
Prevalence of parents' non-intention to vaccinate their children and adolescents against COVID-19 in the regions of Colombia and Peru.

In Colombia, the regions with the highest prevalence of parentś non-intention to vaccinate children against COVID-19 were Amazonas (14.95 %), Putumayo (13.72 %), and Vichada (12.96 %), while the lowest prevalence of non-intention to vaccinate children against COVID-19 was in Vaupés (0 %), San Andrés and Providencia (0.27 %), and Bolivar (5.33 %) **(**Fig. 2**a)**.

#### Bivariate analysis according to parents’ non-intention to vaccinate their children and adolescents against COVID-19 in Colombia

We found statistically significant differences for age, level of education, compliance with physical distancing and use of a mask, economic insecurity, anxiety symptoms, presenting comorbidities, and having been vaccinated **(**Table 2**)**.

**Table 2:** Bivariate analysis of the characteristics of the study sample according to parentś intention to vaccinate their children and adolescents against COVID-19 in Colombia and Peru.

**Characteristics**	**Colombia**							**Peru**						
	**Parents’ intention to vaccinate their children adolescents against COVID-19**							**Parents’ intention to vaccinate their children and adolescents against COVID-19**						
	Yes			No				Yes			No			
	Absolute frequency of participants surveyed	Weighted proportion according to each category		Absolute frequency of participants surveyed	Weighted proportion according to each category		p-value	Absolute frequency of participants surveyed	Weighted proportion according to each category		Absolute frequency of participants surveyed	Weighted proportion according to each category		p-value
	n	%	95 %CI	n	%	95 %CI		n	%	95 %CI	n	%	95 %CI	
**Gender**							0.457							<0.001
Male	17,642	92.75	92.02–93.43	1,291	7.24	6.57–7.98		10,916	92.22	90.61–93.57	800	7.77	6.43–9.39	
Female	23,762	92.44	91.59–93.23	1,983	7.55	6.77–8.41		11,922	94.38	93.44–95.20	664	5.61	4.80–6.56	
**Age (years)**							<0.001							<0.001
18–24	3,698	89.29	87.64–90.75	469	10.7	9.25–12.36		2,642	92.07	89.46–94.09	195	7.92	5.91–10.54	
25–34	12,075	91.10	90.30–91.85	1,194	8.89	8.15–9.70		6,312	91.75	89.95–93.26	481	8.24	6.74–10.05	
35–44	16,056	93.58	92.85–94.24	1,073	6.41	5.76–7.15		7,732	93.98	92.81–94.97	452	6.01	5.03–7.19	
45–54	9,575	94.69	93.79–95.48	538	5.30	4.52–6.21		6,152	94.85	93.95–95.62	336	5.14	4.38–6.05	
**Educational level**							<0.001							0.018
University post-graduate degree completed/university completed/college/pre-university	17,619	93.55	92.97–94.10	1,177	6.44	5.90–7.02		12,505	93.88	92.81–94.80	738	6.11	5.20–7.19	
Secondary school completed/High school (or equivalent) completed	20,294	92.12	91.41–92.78	1,729	7.87	7.22–8.59		9,736	92.96	91.31–94.32	666	7.03	5.68–8.69	
Primary school completed/Less than primary school/No formal schooling	3,491	91.46	89.50–93.09	368	8.53	6.91–10.50		597	90.86	86.78–93.78	60	9.13	6.22–13.22	
**Area of residence**							0.054							<0.001
City	28,051	92.94	92.13–93.67	2,105	7.05	6.32–7.87		18,291	94.09	93.11–94.94	1,049	5.90	5.06–6.89	
Town	9,735	91.90	91.03–92.69	843	8.09	7.31–8.97		2,590	92.41	91.05–93.58	203	7.58	6.42–8.95	
Village or rural area	3,618	92.38	91.17–93.45	326	7.61	6.55–8.83		1,957	89.38	86.70–91.57	212	10.61	8.43–13.30	
**Smoking**							0.104							0.797
No	36,460	92.71	92.08–93.29	2,861	7.28	6.70–7.92		20,071	93.34	92.05–94.44	1,294	6.65	5.56–7.95	
Yes	4,944	91.55	89.70–93.10	413	8.44	6.90–10.30		2,767	93.49	91.80–94.86	170	6.50	5.14–8.20	
**Compliance with physical distancing**							<0.001							<0.001
No	3,850	88.10	86.27–89.73	546	11.89	10.27–13.73		1,817	84.58	81.22–87.44	321	15.41	12.56–18.78	
Yes	37,554	93.11	92.48–93.70	2,728	6.88	6.30–7.52		21,021	94.29	93.21–95.21	1,143	5.70	4.78–6.79	
**Compliance with mask use**							<0.001							<0.001
No	2,801	86.88	85.53–88.13	387	13.11	11.87–14.46		1,203	87.34	82.07–91.23	134	12.65	8.77–17.93	
Yes	38,603	93.06	92.40–93.68	2,887	6.93	6.32–7.60		21,635	93.75	92.70–94.66	1,330	6.24	5.34–7.30	
**Food insecurity**							0.908							0.004
No	12,237	92.61	91.50–93.60	965	7.38	6.40–8.49		5,701	91.85	89.71–93.58	444	8.14	6.42–10.29	
Yes	29,167	92.57	91.97–93.13	2,309	7.42	6.87–8.03		17,137	93.83	92.63–94.85	1,020	6.16	5.15–7.37	
**Economic insecurity**							0.007							<0.001
No	5,996	91.39	89.97–92.62	562	8.60	7.37–10.03		2,341	89.18	86.50–91.39	262	10.81	8.61–13.50	
Yes	35,408	92.77	92.14–93.36	2,712	7.22	6.64–7.86		20,497	93.82	92.62–94.85	1,202	6.17	5.15–7.38	
**Anxiety symptomatology**							0.032							<0.001
No	25,981	92.29	91.48–93.04	2,215	7.70	6.96–8.52		13,108	92.53	90.83–93.95	932	7.46	6.05–9.17	
Yes	15,423	93.10	92.42–93.73	1,059	6.89	6.27–7.57		9,730	94.49	93.52–95.32	532	5.50	4.68–6.48	
**Depressive symptomatology**							0.575							<0.001
No	23,408	92.51	91.76–93.20	1,895	7.48	6.80–8.24		11,389	92.47	90.92–93.78	838	7.52	6.22–9.08	
Yes	17,996	92.68	91.93–93.37	1,379	7.31	6.63–8.07		11,449	94.25	93.12–95.20	626	5.74	4.80–6.87	
**Comorbidities**							<0.001							<0.001
No	26,216	91.72	90.92–92.46	2,328	8.27	7.54–9.08		13,832	92.40	91.02–93.50	1,020	7.59	6.41–8.98	
Yes	15,188	94.05	93.13–94.87	946	5.94	5.13–6.87		9,006	95.12	94.21–95.89	444	4.87	4.11–5.79	
**Vaccinated**							<0.001							<0.001
No	19,559	86.91	85.81–87.94	2,752	13.08	12.06–14.19		12,127	89.71	87.94–91.26	1,213	10.28	8.74–12.06	
Yes	21,845	97.72	97.30–98.08	522	2.27	1.92–2.70		10,711	97.59	97.15–97.96	251	2.40	2.04–2.85	
**Had COVID-19**							0.467							0.002
No	26,834	92.49	91.81–93.13	2,115	7.50	6.86–8.19		12,593	92.61	90.85–94.05	845	7.38	5.95–9.15	
Yes	14,570	92.74	91.88–93.51	1,159	7.25	6.48–8.12		10,245	94.24	93.29–95.08	619	5.75	4.92–6.71	

95 %CI: 95 % Confidence Intervals.

#### Factors associated with parents’ non-intention to vaccinate their children and adolescents against COVID-19 in Colombia

In the adjusted regression model, we found that age groups between 35 and 44 years old (aPR = 0.77; 95 %CI: 0.66–0.90; p = 0.001) and 45 to 54 years old (aPR = 0.78; 95 %CI: 0.65–0.95; p = 0.012) were associated with a lower prevalence of non-intention to vaccinate children against COVID-19 compared to the age group between 18 and 24 years. Likewise, compliance with physical distancing (aPR = 0.55; 95 %CI: 0.49–0.61; p < 0.001), use of masks (aPR = 0.71; 95 %CI: 0.65–0.78; p < 0.001), economic insecurity (aPR = 0.72; 95 %CI: 0.65–0.89; p < 0.001), anxiety symptoms (aPR = 0.87; 95 %CI: 0.79–0.95; p = 0.003), was associated with a lower prevalence of non-intention to vaccinate children against COVID-19. Additionally, having one or more comorbidities (aPR = 0.83; 95 %CI: 0.75–0.91; p < 0.001) and being vaccinated against COVID-19 (aPR = 0.17; 95 %CI: 0.15–0.20; p < 0.001) were associated with a lower prevalence of non-intention to vaccinate children against COVID-19 **(**Table 3**)**.

**Table 3:** Factors associated with parents’ non-intention to vaccinate their children and adolescents against COVID-19 in Colombia and Peru.

**Characteristics**	**Colombia**						**Peru**					
	**Parents’ non-intention to vaccinate their children and adolescents against COVID-19**						**Parents’ non-intention to vaccinate their children and adolescents against COVID-19**					
	Crude			Adjusted			Crude			Adjusted		
	cPR	95 %CI	p-value	aPR	95 %CI	p-value	cPR	95 %CI	p-value	aPR	95 %CI	p-value
**Gender**												
Male	Reference	–	–	Reference	–	–	Reference	–	–	Reference	–	–
Female	1.04	0.93–1.16	0.452	1.06	0.94–1.10	0.318	0.72	0.64–0.81	<0.001	0.81	0.71–0.93	**0.002**
**Age (years)**												
18–24	Reference	–	–	Reference	–	–	Reference	–	–	Reference	–	–
25–34	0.83	0.74–0.93	0.003	0.92	0.82–1.03	0.178	1.04	0.84–1.28	0.703	1.15	0.92–1.43	0.218
35–44	0.60	0.51–0.70	<0.001	0.77	0.66–0.90	**0.001**	0.76	0.61–0.95	0.018	0.92	0.73–1.16	0.488
45–54	0.49	0.40–0.61	<0.001	0.78	0.65–0.95	**0.012**	0.65	0.52–0.81	<0.001	0.96	0.73–0.27	0.776
**Educational level**												
University post-graduate degree completed/university completed/college/pre-university	Reference	–	–	Reference	–	–	Reference	–	–			
Secondary school completed/High school (or equivalent) completed	1.22	1.14–1.32	<0.001	0.95	0.87–1.02	0.165	1.15	1.01–1.31	0.033	0.94	0.83–1.05	0.258
Primary school completed/Less than primary school/No formal schooling	1.32	1.11–1.58	0.003	0.92	0.77–1.09	0.320	1.49	1.07–2.07	0.018	1.06	0.82–1.37	0.635
**Area of residence**												
City	Reference	–	–	Reference	–	–	Reference	–	–	Reference	–	–
Town	1.15	1.02–1.30	0.028	1.09	1.00–1.10	0.060	1.28	1.10–1.50	0.002	1.16	1.00–1.35	**0.048**
Village or rural área	1.08	0.92–1.26	0.331	0.94	0.81–1.00	0.426	1.80	1.51–2.14	<0.001	1.0.51	1.26–1.80	**<0.001**
**Smoking**												
No	Reference	–	–	Not included *			Reference	–	–	Not included *		
Yes	1.16	0.97–1.38	0.095				0.98	0.82–1.16	0.794			
**Compliance with physical distancing**												
No	Reference	–	–	Reference	–	–	Reference	–	–	Reference	–	–
Yes	0.58	0.51–0.66	<0.001	0.55	0.49–0.61	**<0.001**	0.37	0.32–0.42	<0.001	0.45	0.39–0.52	**<0.001**
**Compliance with mask use**												
No	Reference	–	–	Reference	–	–	Reference	–	–	Reference	–	–
Yes	0.53	0.48–0.58	<0.001	0.71	0.65–0.78	**<0.001**	0.49	0.38–0.64	<0.001	0.71	0.56–0.90	**0.005**
**Food insecurity**												
No	Reference	–	–	Not included *			Reference	–	–	Not included *		
Yes	1.01	0.91–1.12	0.907				0.76	0.64–0.90	0.002			
**Economic insecurity**												
No	Reference	–	–	Reference	–	–	Reference	–	–	Reference	–	–
Yes	0.84	0.74–0.95	0.005	0.72	0.65–0.80	**<0.001**	0.57	0.49–0.66	<0.001	0.66	0.59–0.75	**<0.001**
**Anxiety symptomatology**												
No	Reference	–	–	Reference	–	–	Reference	–	–	Reference	–	–
Yes	0.89	0.81–0.99	0.028	0.87	0.79–0.95	**0.003**	0.74	0.64–0.84	<0.001	0.82	0.70–0.97	**0.020**
**Depressive symptomatology**												
No	Reference	–	–	Reference	–	–	Reference	–	–	Reference	–	–
Yes	0.98	0.90–1.06	0.570	1.00	0.91–0.10	0.949	0.76	0.70–0.84	<0.001	0.92	0.82–1.03	0.162
**Comorbidities**												
No	Reference	–	–	Reference	–	–	Reference	–	–	Reference	–	–
Yes	0.72	0.64–0.80	<0.001	0.83	0.75–0.91	**<0.001**	0.64	0.54–0.76	<0.001	0.74	0.63–0.86	**<0.001**
**Vaccinated**												
No	Reference	–	–	Reference	–	–	Reference	–	–	Reference	–	–
Yes	0.17	0.15–0.20	<0.001	0.17	0.15–0.20	**<0.001**	0.23	0.20–0.28	<0.001	0.24	0.20–0.28	**<0.001**
**Had COVID-19**												
No	Reference	–	–	Reference	–	–	Reference	–	–	Reference	–	–
Yes	0.97	0.86–1.06	0.462	0.95	0.87–1.03	0.256	0.78	0.67–0.90	0.001	0.83	0.73–0.94	**0.006**

95 %CI: 95 % confidence intervals; cPR: Crude prevalence ratio; aPR: Adjusted prevalence ratio.

* Not included due to not having a statistically significant association in the crude model.

### Peru

#### Characteristics of the study sample in Peru

In the Peruvian sample, 52.81 % were female, 33.01 % were between 35 and 44 years old, 51.48 % had at least a full university post-graduate degree completed/university completed/college/pre-university, 75.98 % lived in a city, and 11.39 % reported having smoked. In addition, 90.39 % and 93.94 % had complied with physical distancing and the use of a mask, respectively. We found that 76.26 % reported having food insecurity, while 89.95 % reported having economic insecurity. Anxiety and depressive symptoms were reported in 42.31 % and 50.01 %, respectively, and 62.81 % of the participants did not report comorbidities, 53.71 % were not yet vaccinated against COVID-19, and 45.91 % had had COVID-19 at some point. The prevalence of parentś non-intention to vaccinate their children and adolescents against COVID-19 was 6.64 % **(**Table 1**)**.

#### Prevalence of parents’ non-intention to vaccinate their children and adolescents against COVID-19 according to each region of Peru

In Peru, the regions with the highest prevalence of non-intention to vaccinate children against COVID-19 were Moquegua (13.73 %), Madre de Dios (13.65 %), and Puno (11.83 %). On the other hand, the regions with a lower prevalence of non-intention to vaccinate children against COVID-19 were Pasco (4.10 %), Lima (4.82 %), and Piura (5.03 %) **(**Fig. 2**b)**.

#### Bivariate analysis according to parents’ non-intention to vaccinate their children and adolescents against COVID-19 in Peru

We found statistically significant differences between the independent variables and the intention of parents to vaccinate their children and adolescents against COVID-19, except for smoking (p = 0.797) **(**Table 2**)**.

#### Factors associated with parents’ non-intention to vaccinate their children and adolescents against COVID-19 in Peru

In the adjusted regression model, we found female gender (aPR = 0.81; 0.71–0.93; p = 0.002) compared to male gender was associated with a lower prevalence of non-intention to vaccinate children against COVID-19. Likewise, compliance with physical distancing (aPR = 0.45; 95 %CI: 0.39–0.52; p < 0.001), use of masks (aPR = 0.71; 95 %CI: 0.65–0.90; p = 0.005), economic insecurity (aPR = 0.66; 95 %CI: 0.59–0.75; p < 0.001), anxiety symptoms (aPR = 0.82; 95 %CI: 0.70–0.97; p = 0.020) were associated with a lower prevalence of non-intention to vaccinate children against COVID-19. In addition, having one or more comorbidities (aRP = 0.74; 95 %CI: 0.63–0.86; p < 0.001), being vaccinated against COVID-19 (aPR = 0.24; 95 %CI: 0.20–0.28; p < 0.001) and having had COVID-19 (aPR = 0.83; 95 %CI: 0.73–0.94; p = 0.006) were associated with a lower prevalence of non-intention to vaccinate children against COVID-19. On the other hand, living in a town (aPR = 1.16; 95 %CI: 1.00–1.35; p = 0.048) and living in a village or rural area (aPR = 1.51; 95 %CI: 1.26–1.80; p < 0.001) compared to living in the city was associated with a higher prevalence of non-intention to vaccinate children against COVID-19 **(**Table 3**)**.

## Discussion

Our main results show that about 9 out of 10 parents in Colombia and Peru intend to vaccinate their children and adolescents against COVID-19. In Colombia, being between 35 and 54 years old, adherent to maintaining physical distance, using masks, having economic insecurity, having symptoms of anxiety and comorbidities, and being vaccinated were associated with a higher probability of vaccinating children against COVID-19. In Peru, being female, an adherent to maintaining social distancing, using a mask, having economic insecurity, having symptoms of anxiety and comorbidities, having been vaccinated, and having had COVID-19 was associated with a lower probability of not having the intention to vaccinate children. On the contrary, living in a town or rural area was associated with a greater intention not to vaccinate.

Our results of the intention to vaccinate children and adolescents are similar to those found in the evaluation carried out between May and June 2021 in LAC [13]. This are good news in terms of public health because the Ministries of Health of Peru and Colombia have already scheduled or started vaccination against COVID-19 with different vaccines in children and adolescents [28], [29]. This acceptance is expected to be reflected in the increase in population coverage of the vaccine to achieve the rate needed to obtain herd immunity. However, variations between the regions of the countries and in the case of Peru, according to the place of residence, require individualization of the vaccination programs based on the region.

As in other LAC countries, some factors associated with the socioeconomic and psychological consequences of the pandemic reduce the non-intention of vaccination[13]. Indeed, aspects such as stress[13], economic or food insecurity as a result of the economic crisis during the pandemic and especially during the first wave [30], possibly created a state of alert and the desire for such a situation not to be repeated, being vaccination seen as an opportunity to achieve this [13]. Along the same line, adherence to community mitigation measures such as the use of masks and social distancing may reflect a higher likelihood of complying with vaccination. Similarly, the feeling of vulnerability [26] and the desire to avoid this happening to their children may foster the positive view of vaccination and reduce the probability of non-intention to vaccinate their children [13].

While some factors coincide among the different countries, some studies have shown that some factors can be explained by social determinants and socioeconomic differences between countries [19], [20], [21], [22], [23], [24]. Although, to date, no study has compared these differences, there may be several explanations. In a previous study by our group evaluating food insecurity in LAC in the first stage of the pandemic, it was found that food insecurity was higher in Peru than in Colombia (83.9 % vs 76.8 %, respectively), which may explain the results of the present study [30]. Similarly, the impact of the pandemic was different in the two countries, with the number of deaths registered until November 17, 2021, being more than 200 thousand in Peru [31] and about 128 thousand in Colombia [32]. These differences might suggest that the fear of having had the infection and its consequences have led to the desire of this not happening to their children and an increase in the intention to vaccinate them. On the other hand, according to the document “Distinctive features of health systems in the world, 2017″ the Colombian health system is better developed than the Peruvian [33]. Indeed, in Peru, despite the improvements, structural problems and coverage of health services in areas such as rural areas had a great impact on health care during the pandemic and could explain our present results [34], [35].

There were great variations in the intention to vaccinate among the different regions of Peru. The regions with the least intention to vaccinate were Madre de Dios, Puno, and Moquegua. In contrast, the regions of Lima and Lambayeque had the highest intention to vaccinate children and adolescents against COVID-19. The Madre de Dios and Moquegua regions presented the lowest fatality during the pandemic, which may explain the lesser feeling of fear, commented previously compared to Lambayeque, for example, which is one of the departments with the highest fatality [31]. However, the complexity of the impact of the pandemic in each region is far from being fully understood, which could explain why, in the department of Puno, a region with low mortality, the intention to vaccinate is low. However, it is likely that some aspects such as self-medication [36], knowledge about the disease [37], and the reliability of the sites where Peruvians obtain information [38] on the pandemic or the variation in prevention practices among Peruvians [39], may explain the differences in intention to vaccinate children among the different regions of Peru.

In Colombia, the departments with the highest vaccination intention were Vaupés, San Andrés, Bolívar, Guainía, and Cundinamarca, being above 94 %. Several of these regions were significantly affected by COVID-19, especially in terms of concentration of cases per inhabitant. In departments such as San Andrés, there was deep state intervention in different aspects due to the initial level of involvement of COVID, which might influence the subsequent vaccination intention. Likewise, in the department of Bolívar, where the capital, Cartagena, is located, there were also a considerable number of cases and deaths, which may influence the intention of vaccination [40]. The vaccination program in Colombia is led by a mass media campaign [41], having the support of scientific societies, such as the Colombian Association of Infectious Diseases, which promotes information based on evidence to both health personnel and the community, and this could also contribute to the intention of vaccination [42].

Our study has some limitations. Since the respondents were users of a social network, information was only obtained from people with access to the internet and social networks, which could vary between the regions of the countries evaluated and the rural population. We do not have the non-response rate, which is relevant in the context of an online survey. The variables included in this analysis were pre-established in the survey, and there could be relevant variables not included in our analysis. The data were obtained by self-reporting and, therefore, an underreporting of information is possible. Finally, due to the design of the study, our results should only be interpreted in the context of associations since causality among the variables evaluated could not be established. However, this study presents the strength of analyzing a database with a large representative sample of social network users widely used in Colombia and Peru.

## Conclusion

In conclusion, about 9 out of 10 parents in Colombia and Peru intend to vaccinate their children and adolescents against COVID-19. This intention was associated with some factors which are similar between the two countries, as well as other factors and variations among the different regions of each country. This means that despite good general acceptance of vaccination against COVID-19, the health authorities of each country must propose individualized strategies depending on the context if the vaccination campaigns in this population group are to achieve their objectives. This includes not only broadening the target groups of both unvaccinated and vaccinated but also achieving high coverage with an additional booster or complementary doses. Finally, scientific evidence must be communicated to the public, including aspects that may affect confidence in vaccines, such as the new variants of concern, including the Omicron variant, among other aspects.

**Declaration of interests**.

Vicente A. Benites-Zapata reports article publishing charges was provided by San Ignacio de Loyola University.

**Competing interests**: The authors declare that they have no competing interests.

**Funding**: This research received no specific grant from any funding agency in the public, commercial, or not-for-profit sectors.

**Authors' contributions**: All authors made substantial contributions to the study's conception and design, analysis and interpretation.

## Declaration of Competing Interest

The authors declare that they have no known competing financial interests or personal relationships that could have appeared to influence the work reported in this paper.
