# Supplementary material for: Prevalence of parents' non-intention to vaccinate their children and adolescents against COVID-19: A comparative analysis in Colombia and Peru
Source: Vaccine X. 2022 Jul 27;12:100198. doi: 10.1016/j.jvacx.2022.100198 (PMC9344873; doi:10.1016/j.jvacx.2022.100198)
Supplement: Supplementary data 1 [file mmc1.docx]

**Supplemental Material**

| **Table S1. Proportion of parents´ intention to vaccinate children and adolescents against COVID-19 according to regions in Colombia.** | | | | |
| --- | --- | --- | --- | --- |
|  | **Colombia** | | | |
|  | **Parents´ intention to vaccinate children and adolescents against COVID-19** | | | |
|  | Yes | | No | |
|  | Weighted proportion | | Weighted proportion | |
| **Region** | % | 95%CI | % | 95%CI |
| Vaupés | 100 | - | 0 | - |
| San Andrés y Providencia | 99.73 | 98.11-99.96 | 0.27 | 0.04-1.89 |
| Bolivar | 94.67 | 93.58-95.58 | 5.33 | 4.42-6.42 |
| Guainía | 94.29 | 68.66-99.20 | 5.71 | 0.80-31.34 |
| Cundinamarca | 94.18 | 93.15-95.06 | 5.82 | 4.94-6.85 |
| Caldas | 93.98 | 91.61-95.70 | 6.02 | 4.29-8.39 |
| Antioquia | 93.86 | 93.08-94.56 | 6.14 | 5.44-6.92 |
| Sucre | 93.72 | 91.69-95.28 | 6.28 | 4.72-8.31 |
| Bogotá | 93.37 | 92.79-93.90 | 6.63 | 6.10-7.20 |
| Nariño | 93.2 | 91.48-94.60 | 6.8 | 5.40-8.51 |
| Córdoba | 93.16 | 91.50-94.52 | 6.84 | 5.48-8.50 |
| Quindío | 93.16 | 90.77-94.97 | 6.84 | 5.03-9.23 |
| Atlántico | 92.93 | 91.80-93.91 | 7.07 | 6.08-8.20 |
| Guaviare | 92.84 | 81.98-97.36 | 7.16 | 2.64-18.02 |
| Chocó | 92.65 | 86.29-96.19 | 7.35 | 3.81-13.71 |
| La Guajira | 92.6 | 90.14-94.47 | 7.4 | 5.53-9.86 |
| Boyacá | 92.54 | 90.49-94.18 | 7.46 | 5.82-9.51 |
| Magdalena | 92.28 | 90.47-93.77 | 7.72 | 6.23-9.53 |
| Cesar | 92.1 | 90.31-93.58 | 7.9 | 6.42-9.69 |
| Risaralda | 91.86 | 89.83-93.52 | 8.14 | 6.48-10.17 |
| Arauca | 91.71 | 87.87-94.41 | 8.29 | 5.59-12.13 |
| Norte de Santander | 91.64 | 90.01-93.03 | 8.36 | 6.97-9.99 |
| Tolima | 91.31 | 89.48-92.86 | 8.69 | 7.14-10.52 |
| Cauca | 91.13 | 89.20-92.73 | 8.87 | 7.26-10.80 |
| Santander | 91.13 | 89.92-92.20 | 8.87 | 7.80-10.08 |
| Valle del Cauca | 90.62 | 89.62-91.53 | 9.38 | 8.47-10.38 |
| Casanare | 90.5 | 87.53-92.82 | 9.5 | 7.18-12.47 |
| Caquetá | 90.4 | 86.77-93.12 | 9.6 | 6.88-13.23 |
| Huila | 89.62 | 87.10-91.69 | 10.38 | 8.31-12.90 |
| Meta | 89.01 | 86.75-90.92 | 10.99 | 9.08-13.25 |
| Vichada | 87.04 | 65.33-95.99 | 12.96 | 4.01-34.67 |
| Putumayo | 86.28 | 81.23-90.14 | 13.72 | 9.86-18.77 |
| Amazonas | 85.05 | 74.49-91.72 | 14.95 | 8.27-25.51 |
| 95%CI: 95% Confidence Intervals | | | | |

| **Table S2. Proportion of parents´ intention to vaccinate children and adolescents against COVID-19 according to regions in Peru.** | | | | |
| --- | --- | --- | --- | --- |
|  | **Peru** | | | |
|  | **Parents´ intention to vaccinate children and adolescents against COVID-19** | | | |
|  | Yes | | No | |
|  | Weighted proportion | | Weighted proportion | |
| **Region** | % | 95%CI | % | 95%CI |
| Pasco | 95.9 | 89.93-98.39 | 4.1 | 1.61-10.07 |
| Lima | 95.18 | 94.72-95.61 | 4.82 | 4.39-5.28 |
| Piura | 94.97 | 93.64-96.04 | 5.03 | 3.96-6.36 |
| Callao | 94.4 | 92.94-95.57 | 5.6 | 4.43-7.06 |
| Lambayeque | 94.18 | 92.41-95.55 | 5.82 | 4.45-7.59 |
| Ancash | 93.95 | 92.22-95.32 | 6.05 | 4.68-7.78 |
| Ica | 93.9 | 92.04-95.35 | 6.1 | 4.65-7.96 |
| Huancavelica | 93.75 | 88.51-96.69 | 6.25 | 3.31-11.49 |
| La Libertad | 93.68 | 92.07-94.97 | 6.32 | 5.03-7.93 |
| Cajamarca | 93.42 | 91.22-95.10 | 6.58 | 4.90-8.78 |
| Junín | 93.09 | 91.27-94.55 | 6.91 | 5.45-8.72 |
| Huánuco | 92.61 | 88.44-95.36 | 7.39 | 4.64-11.56 |
| Arequipa | 92.26 | 90.75-93.55 | 7.74 | 6.45-9.25 |
| Tacna | 92.16 | 88.59-94.69 | 7.84 | 5.31-11.41 |
| Loreto | 92.14 | 88.83-94.52 | 7.86 | 5.48-11.17 |
| Ayacucho | 91.61 | 87.01-94.68 | 8.39 | 5.32-12.99 |
| Apurímac | 91.56 | 84.85-95.45 | 8.44 | 4.55-15.15 |
| Cusco | 91.43 | 89.22-93.21 | 8.57 | 6.79-10.78 |
| Amazonas | 91.25 | 86.37-94.49 | 8.75 | 5.51-13.63 |
| Ucayali | 90.78 | 87.22-93.43 | 9.22 | 6.57-12.78 |
| San Martín | 89.95 | 86.78-92.43 | 10.05 | 7.57-13.22 |
| Tumbes | 89.85 | 81.34-94.73 | 10.15 | 5.27-18.66 |
| Puno | 88.17 | 84.92-90.79 | 11.83 | 9.21-15.08 |
| Madre de Dios | 86.35 | 78.93-91.44 | 13.65 | 8.56-21.06 |
| Moquegua | 86.27 | 71.79-93.94 | 13.73 | 6.06-28.21 |
| 95%CI: 95% Confidence Intervals | | | | |
